# Addressing barriers to post-graduate research training in low resource settings: an innovative approach in an institution of higher learning in Kenya

**DOI:** 10.3389/fmed.2024.1470922

**Published:** 2024-11-13

**Authors:** Eunice Muthoni Mwangi, Rosebella Alungata Iseme-Ondiek, Roselyter Monchari Riang'a, James Orwa, Kennedy W. Njenga, Anthony K. Ngugi

**Affiliations:** ^1^Department of Population Health, Medical College, Aga Khan University, Nairobi, Kenya; ^2^Post Graduate Medical Education, Medical College, Aga Khan University, Nairobi, Kenya

**Keywords:** dissertation, curriculum, postgraduate, research methods, resident, innovation in training, research milestones, longitudinal approach

## Abstract

**Background:**

Numerous barriers hinder health research training in Post-Graduate Medical Education Programs, especially in developing countries. Research training is pivotal for medical residency, providing essential knowledge and skills for dissertation completion, fostering evidence-based medical practice, and nurturing future independent clinical researchers. A holistic approach to research education is imperative to surmount these barriers. We describe here a dissertation-centric research curriculum, delivered longitudinally, an innovative strategy undertaken by Aga Khan University's Medical College in East Africa (AKU-MCEA) to deliver postgraduate research.

**Methods:**

A review of AKU-MCEA post-graduate research methods curriculum was conducted based on implementing Departments' experience, institutional policies, and residency program output.

**Program implementation:**

The Master of Medicine program requires a well-executed dissertation for graduation. Residents undergo structured research training across four modules, with interactive sessions and workshops supporting their research milestones. Protected time allows residents to develop and report on their dissertation work. Faculty receive specialized training in dissertation supervision to ensure residents benefit from expert guidance in producing high-quality research.

**Outcomes:**

This approach achieves a dissertation completion and graduation rate of 98–100%. Notably, 68.5% (*n* = 126) of those who graduated in 2013–2023 have published two or more papers (i.e., range 2–36) in their current portfolio, demonstrating a commitment to ongoing research endeavors even after graduation. Residents' research covers diverse themes and is disseminated through departmental presentations, university-wide Faculty Academic Rounds, peer-reviewed journals, conferences, and the Annual Early Career Researchers symposium.

**Lessons learnt:**

A comprehensive approach featuring a structured research curriculum, longitudinal delivery with predetermined milestones, dedicated supervision, financial and resource support, protected research time, ongoing faculty development in dissertation supervision, and clear institutional research policies accelerates dissertation completion and enhances effective research dissemination.

## Introduction

Residency training programs are expected to allow residents to be exposed to research (1). Health research has a central role in improving the health of individuals and populations, which is the professional responsibility of all doctors and medical students striving to specialize within the medical profession. Notably, physicians are uniquely positioned to translate their experience into research investigations through their daily interaction with patients, which may result in positive patient outcomes. Furthermore, whether or not a doctor pursues a research career, they still need to be able to critically review work undertaken by others as a means of validating the content and relevance to their context (2), and in the application of evidence-based medicine. The knowledge and utility of research skills are therefore imperative. As such medical students need to be provided with a foundation necessary to develop their own rigorous and informed approaches to research and critical review of evidence through training.

Low-resource settings encompasses any environment where resources may be limited in terms of quantity, quality, or both, across time and space (3). Several barriers impede health research training in many Post-Graduate Medical Education Programs (PGME), particularly in developing countries. These include, among others, both faculty and students being unfamiliar with the institutional research policies and guidelines, long and tedious proposal approval and ethics review processes, lack of enforcement in supervision load limit (4), lack of a dedicated research curriculum (5–7), inadequacy of research faculty, underdeveloped research culture, competition for time between clinical expectations and research (5, 7), inadequate supervision, guidance and mentorship, and limited financial (5, 7–10), and other resources (2). The challenges contributing to failure by postgraduates to complete dissertation research on schedule are related to inadequacies of the supervisors, of the academic institutions, and students' personal and academic problems (11–13).

Lack of dedicated research curricula is consistently highlighted in academic literature as a significant obstacle, impeding the progress and timely completion of postgraduate medical education (5–7). The quality and relevance of curricula in health training institutions, including medical schools, are also found to be inadequate, failing to prepare students for clinical placement and national health needs (14). These studies indicate that trainings in most institutions with some level of postgraduate research are largely theoretical, running over a term or semester, this is likely to lead to poor learning outcomes, delays in completing postgraduate studies, frustrations and negative attitudes toward research, unethical conduct of research and low conversion of dissertations into publications, mainly because publication is not a mandatory requirement (9). These challenges hinder the development of research skills among students and impede the generation of knowledge crucial for addressing local and global health issues.

In East Africa, where higher education institutions face similar constraints particularly in relation to curriculum design and delivery, innovative approaches are essential to overcome these barriers and promote scholarly excellence. We describe such an approach, developed, and implemented within the residency program at Aga Khan University's twin Medical College (AKU-MCEA) campuses in Nairobi and Dar es Salaam, East Africa, that is, a Dissertation-Centric postgraduate research curriculum which is delivered longitudinally in tandem with expected research milestones, along with the creation of a supportive research environment. This manuscript outlines this unique curriculum approach, its implementation, and outcomes with specific focus on the governing policies, regulations and practices underlying successful implementation, offering valuable insights for educators and institutions seeking to enhance research training and implementation at the post-graduate level.

## Methods

Review of the AKU-MCEA post-graduate Research Methods curriculum was conducted based on implementing Departments' experiences, institutional policies, and residency program output.

### Participants in the research methods course

The Research Methods course is a common and compulsory course for residents in all nine Master of Medicine (MMed) programs at AKU: Anesthesiology, Anatomic Pathology, Clinical Pathology, Family Medicine, General Surgery, Imaging and Diagnostic Radiology, Internal Medicine, Obstetrics and Gynecology, and Pediatrics and Child Health. All residents enter AKU's postgraduate residency program, earn a MMed degree upon satisfactory fulfillment of the training requirements. Applicants must possess a basic degree in medicine and are selected on merit through a competitive process that includes an entrance exam and panel interviews where scoring follows a standardized rubric. Residents pay a nominal annual tuition fee and receive a stipend and a research grant for their studies. Training occurs at AKU Hospitals in Nairobi Kenya and Dar es Salaam Tanzania, both tertiary private facilities, where the residency and MMed program run concurrently. Both programs are accredited by the Higher Education regulators and Medical Councils in the two countries. Female residents comprise 46.8% and males 53.2%.

## Results

### Curriculum development

The postgraduate research methods curriculum adopted by AKU-MCEA responds to the needs identified in practice and policy. Based on needs assessment undertaken in 2018, clear, measurable learning objectives were defined, and a revised curriculum was implemented in 2019. Needs assessment was undertaken among faculty (*n* = 35), alumni (*n* = 65), and residents (*n* = 105). Purposive selection was done to represent all faculty who had provided supervision and alumni who had completed the program as well as residents who were active at the time of the survey. The assessment revealed that 88.9% of faculty were dissatisfied with residents' competency in conducting publishable research, with an average of only 20% achieving this standard. Additionally, 89.9% of faculty felt the time allocated for research was insufficient, and only 43.8% of residents and 27.3% of alumni reported feeling confident in undertaking independent research. Notably, 93.7% of residents expressed a need for more time dedicated to the course. The assessment highlighted several issues with the previous curriculum, including limited credit hours relative to content, a focus on theoretical instruction with minimal practical skills training, inadequate research funding, and ongoing challenges for residents in completing their dissertations. Furthermore, there was insufficient time to explore topics in depth, and the curriculum lacked continuous supportive supervision and monitoring. Key recommendations for improvement include redesigning the research curriculum to deliver the course longitudinally over 4 years, minimize didactic sessions, allocate more time for practical activities like data analysis, provide residents with additional research time, and offer funding to support research initiatives. These recommendations were thereafter used to improve the curriculum creating more time and making it more practical, with implementation being longitudinal and tied to specific research milestones.

The curriculum undergoes review every 5 years as recommended by the Commission for University Education (CUE), aimed at ensuring that it is fulfilling its purpose and that it is responsive to emerging trends, changing student needs and an ever-evolving research field. Review of the 2019 curriculum is underway. In addition, regular evaluation of the effectiveness of the curriculum is undertaken through residents' surveys, course evaluation through the Virtual Learning Environment (VLE) platform and outcomes assessment. The information generated from these evaluations is used to guide any adjustments and enhancements to the delivery of the curriculum and this goes a long way in improving the curriculum and in meeting the expected outcomes.

### Curriculum content

The research methods curriculum aims at enhancing the ability of residents to develop a research question, undertake a systematic literature search, design a research study, collect and manage data, conduct data analysis, write a research paper, and disseminate findings in different forums. The curriculum guides delivery of a research methods course that is divided into four thematic areas delivered in four modules. The implementation roadmap is overseen by the office of Postgraduate Medical Education (PGME), while content delivery is led by the Department of Population Health (DPH).

The first of the four thematic areas, Introduction to Research and Ethical Conduct of Research, is aimed at providing a broad introduction to the concepts and principles of research, ethical conduct of research using human subjects and to impart skills necessary for the development of research questions and scientific writing. The second thematic area is Introduction to Research Methods, whose purpose is to introduce epidemiology by exposing residents to the principles, methodologies, uses, and application of epidemiological methods in biomedical, clinical, and population health research. The third thematic area is Introduction to Qualitative Research, which aims to introduce residents to the field of qualitative research and to equip them with knowledge, skills, and techniques necessary to undertake independent research using this methodology. The fourth thematic area is Biostatistics in health research. The purpose of this final thematic area is to introduce residents to biostatistical approaches by exposing them to the principles, methodologies, uses, and applications of statistical methods in biomedical and clinical research (see Table 1).

**Table 1:** Research methods course content and implementation schedule.

**Module**	**Year of study**	**Topics**	**Teaching method**	**Total contact time (h)**	**Taught h**	**Workshop h**	**Expected outcome**
Introduction to research and ethical conduct of research	1st	Ethical conduct of research	Online self-study (CITI)		NIL	NIL	Fully formed research question and 2-page concept paper
		Formulating research questions	Interactive lecture and practical	22.5	1	2	
		Sources of and acquisition of literature	Interactive lecture and practical		1	2	
		Critical appraisal of healthcare literature	Interactive lecture and practical		1	2	
		Introduction to summarizing evidence	Interactive lecture and practical		1	1	
		Introduction to scientific writing	Interactive lecture and practical		1	2	
		Writing for publication I	Interactive lecture and practical		1.5	1	
		Steps in the research process	Interactive lecture		1.5	0	
Introduction to research methods	1st	Introduction to epidemiologic principles	Interactive lecture		1	0	Fully developed dissertation proposal
		Measurement of disease and health in populations	Interactive lecture and practical		1.5	2	
	2nd	**Introduction to common research designs**					
		Descriptive studies—case reports/series, and descriptive and analytic cross-sectional studies	Interactive lecture	44	2.5	0	
		Case-control and cohort studies	Interactive lecture		2	0	
		Experimental/intervention studies	Interactive lecture		1.5	0	
		Diagnostic and screening test utility studies	Interactive lecture and practical		1.5	2	
		Screening, epidemiologic surveillance and outbreak investigation	Interactive lecture		2	0	
		Introduction to systematic reviews	Interactive lecture and practical		2	2	
		**Issues in epidemiologic studies**					
		Measurement error (validity and reliability), Selection and information bias	Interactive lecture		2.5	0	
		Confounding and interaction	Interactive lecture		2	0	
		Causality	Interactive lecture and practical		1.5	2	
		**Introduction to Biostatistics**					
		Definition and interpretation of common statistical tests	Interactive lecture		2	0	
		Introduction to power and sample size calculations	Interactive lecture and practicals		2.5	3	
		**Introduction to qualitative research**					
		Qualitative vs. quantitative research	Interactive lecture		0.5	0	
		Qualitative research designs	Interactive lecture		0.5	0	
		Sampling in qualitative research	Interactive lecture		1	0	
		Qualitative research methods			2	0	
		Qualitative research methods	Interactive lecture and practical		3	4	
		Writing for publication II	Interactive lecture and practical		1	1	
Biostatistics in health research/qualitative analysis	3rd	**Statistical methods/qualitative analyses**					Analyzed data and dissertation results section
		Descriptive statistics	Interactive lecture and practical	30	1.5	1.5	
		Students' *t*-test, chi squared tests, correlation and linear regression	Interactive lecture and practical		1.5	2	
		Logistic regression, Poisson regression	Interactive lecture and practical		2.5	2	
		General linear models, Kaplan Meier and cox regression models	Interactive lecture and practical		2.5	2	
		Data management practices	Interactive lecture and practical		2.5	2	
		Analyzing qualitative data	Interactive lecture and practical		2	3	
		Analyzing qualitative data	Interactive lecture and practical		1	4	
Writing support	3rd and 4th	Editorial consultation			Open	Open	Full dissertation and manuscript
Total hours				96.5	54	42.5	

### Curriculum implementation

AKU-MCEA integrates a Dissertation-centric Research Curriculum within its 4-year Master of Medicine (MMEd) program, spanning nine specialties (15). The mandatory Research Methods course equips residents to design, implement, and report on their research dissertations, fulfilling graduation requirements. The curriculum progresses from foundational to advanced topics, fostering skill development and ethical research conduct tailored to each resident's study area within the institutional guidelines. Dissertation work spans all 4 years of training (15). Each year of training has set research milestones: a focused research question and concept paper by end of year one; a fully developed proposal by end of year two; analyzed data integrated into the dissertation by end of year three; and a completed dissertation and manuscript ready for journal submission by first quarter of year four. The four thematic areas are covered in research modules totaling 96.5 contact hours (54 taught hours and 42.5 workshop hours), as shown in Table 1.

Residents are required to complete the Collaborative Institutional Training Initiative (CITI) course on research ethics upon enrollment. In the first quarter of year one, residents collaborate with faculty in their department to identify research questions, followed by formal research methods sessions in the second quarter of year one.

The first module, *Introduction to research and ethical conduct of research*, is covered in the second half of year one over 22.5 contact hours (10.5 taught hours and 12 faculty guided workshop hours). The didactic components of the course are complemented with aligned faculty guided workshops in which residents work individually, in small groups and in the plenary to apply the theory learnt to their actual research topics. Each teaching and workshop session lasts on average 2 h. Residents are expected to develop a 2-page concept paper as a key deliverable at the end of the module.

Module two is covered over a duration of 44 contact hours (30 taught hours and 14 faculty guided workshop hours). Didactic interactive sessions cover topics such as introduction to common research designs and issues in epidemiology. Each taught and workshops session lasts between 1 and 4 h. Residents are expected to produce a full dissertation proposal for submission to the Institutional Scientific Ethics and Review Committee (ISERC) as a deliverable. After approval of protocol by various regulatory bodies, data collection commences late in the second year or early in the third year of training.

The third module, *Biostatistics in health research and qualitative analysis* is covered in the second half of year 3. The module is covered over a period of 30 contact hours (13.5 taught and 16.5 workshop hours) systematically spread out across the third year. At the end of the third year, the resident is expected to have completed analyzing their data and drafted a dissertation results section.

The fourth module, *writing support*, is offered in the second half of year 3 and first half of year 4 of study. This is done through editorial support offered by professional editors at the university library alongside the content and methodology supervisors. Residents are supported to write up their results and complete their full dissertation as well as produce a manuscript for publication. A dissertation should be ready in the first quarter of year four of residency. The resident is also expected to develop a manuscript for submission to a peer reviewed journal. Manuscript submission follows final dissertation submission for examination. Students are required to disseminate the final work through conferences, symposia, and other approved forums, such as the weekly university wide Faculty Academic Rounds (FARs) and the Annual Early Career Researchers symposium (AECRS).

### Curriculum delivery strategies, governing principles, regulations, and practices

Successful implementation of the research methods curriculum at AKU may be attributed to several principles and practices. Firstly, the postgraduate research policy and guidelines are disseminated to the residents during orientation to the MMed program with an aim of setting out the expected research milestones. The research curriculum is thereafter delivered longitudinally over a period of 4 years in tandem with expected research milestones.

The delivery of the curriculum is undertaken by multiple health professionals with dynamic experience in epidemiology, biostatistics, qualitative methods, and critical writing skills. Furthermore, each resident is assigned 2–4 experienced methodological and content supervisors to provide guidance, review research proposals, offer methodological expertise, and facilitate networking opportunities. Notably, faculty and staff undergo capacity development in dissertation supervision and research, under the stewardship of more experienced MCEA Faculty, led by the DPH with support from other departments, among which include the Academic Office, the Research Office, AKU Library and Grants Office.

The research curriculum is implemented though a blended format, where content is delivered online via Zoom, with recorded sessions available on the university's VLE platform and face-to-face didactic sessions. This approach combines synchronous and asynchronous learning, as well as streamline data collection, analysis, and dissemination in training of residents.

The delivery of the curriculum occurs through interactive didactic lecturers, workshops, seminars, and practical training. Hands-on training sessions using research tools and software relevant to the respective residents' research method/design is also undertaken. Varied assessment methods at each stage, including oral presentations, written reports, and peer reviews are utilized through an established feedback loop where residents receive constructive feedback from mentors, peers, and faculty members. These strategies are intended to promote continuous improvement.

Residents have unfettered access to critical research infrastructure and a comprehensive set of resources, including textbooks, journals, online databases, and software tools, tailored to residents' research domains are provided by the AKU Library. Residents are guided by professionally trained librarians on how to search for physical reference materials, and how to use Artificial Intelligence (AI) techniques in literature review. The MCEA provides each resident with research support of USD $1,000 upon successful application, protected research time of 8 weeks over the 4 years for successful dissertation completion, on-demand research methodology and scientific writing consulting clinics. Quality assurance mechanisms have been put in place to ensure quality dissertation and research outcomes.

### Curriculum implementation outcome

Implementation of the Dissertation-Centric research curriculum longitudinally in line with expected research milestones has been critical in addressing the barriers to post-graduate research training and promote a culture of scholarly excellence. The first cohort of residents was enrolled in 2004. Intake into the program happens annually. As of September 2023, a total of 552 residents had been successfully enrolled into the PGME training program, out of which 145 are still in the early/mid stages of training. Out of 407 residents, 363 (89.2%) have successfully completed their training with the remaining 10.8% dropping off from the program due to personal/non-academic reasons. Notably, dissertation completion is one of the prerequisites for graduation, six out of 363 (1.7%) graduated a year after their scheduled date due to a delay in completing their dissertation. This in stark contrast to delayed completion rates reported in other Health Science institutions within the region. For instance, a study in Makerere University College of Health Sciences reported a delayed dissertation completion rate of 82.2% (16). The demand for, and enrollment into the AKU-MCEA residency program has been on an upward trend, with more than 70% increase in demand for the programs since 2017.

While publication is not a mandatory requirement for completing the MMed program, the Commission for University Education strongly recommends that all graduate students, regardless of their program, publish at least one article in a refereed journal before graduation. Consequently, this practice is highly encouraged among all residents in the AKU-MCEA program. In line with this, students are noted to publish one academic paper during their program of study. From 2013 to 2023, of the 331 residents who graduated from the MMed program, more than half (*n* = 184; 55.6%) successfully published their graduate research with 240 publications to date. Notably, 68.5% (*n* = 126) of those who published have two or more papers (i.e., range 2–36) in their current portfolio, demonstrating a commitment to ongoing research endeavors even after graduation. At present a total of 17 alumni have published over 10 papers whilst 12 have more than 20 publications.

## Discussion

The principles and practices supporting residents' timely completion of dissertation over the years can be broadly categorized as institutional, personal and supervisor related factors.

### Institutional related factors

Institutional research capacity strengthening mechanisms are associated with having in place method of formal training, promoting a research-conducive environment and establishment of research support systems (17). Aga Khan University focuses on advancing global knowledge and shaping public policy for developing communities. It cultivates a strong research culture, supporting students and faculty. Over the years faculty and residents from the MCEA have published 3,258 papers, with a total of 171,660 downloads as of September 2024, indicating substantial research output (18). Residents are often engaged as junior researchers and co-investigators in several ongoing research projects in the MCEA. Linking students training to ongoing research can enhance student research, accelerate their training process, and link them to a global network of scholars (19).

#### Dedicated research methods curriculum

Implementing a longitudinal as well as dedicated multifaceted research curriculum significantly increases residents' research skills, satisfaction and participation in scholarly activities including dissemination of findings at regional and national forums (20, 21). Some learning institutions lack a dedicated research curriculum, and for those with some level of research training, these trainings are largely theoretical running as a block delivered over a term or semester, leading to poor research outcomes.

A longitudinal research curriculum, incorporating interactive didactic sessions, mentored workshops, and plenary sessions, enhances residents' research skills, expands their expertise in specific areas, and promotes critical thinking. This approach encourages residents to analyze complex issues, synthesize information, identify knowledge gaps, and develop original research questions, thereby fostering publication and dissemination of research findings. This approach often includes research-focused lectures, journal clubs, clear project timelines, and dedicated research time within the residency schedule (20–22).

#### Scientific ethical review and postgraduate guidelines

Ethics review procedures mandated by academic and research institutions require researchers to seek approval from ethics boards before conducting research, aiming to ensure ethical standards. However, criticism exists regarding the bureaucratic and sometimes obstructive nature of these boards (23). Most institutions have postgraduate research guidelines, yet these are often inaccessible, hindering consistency and timely dissertation completion. There is a call for improved transparency and communication of ethical review timelines to mitigate research delays (24).

The AKU postgraduate research policies and guidelines have clearly spelt out milestones (15), these guidelines are available in the university website and are disseminated to faculty and staff and to the residents during orientation to postgraduate programs. In addition, AKU-ISERC circulates the annual schedule of its activities including ethical review timelines, information that is important for researchers to plan ahead (25). This facilitates timely ethical review of protocols.

#### Interprofessional collaboration in curriculum delivery

The residency research curriculum at AKU-MCEA involves multiple health professionals collaborating to train residents, this approach offers diverse perspectives and expertise across healthcare disciplines, optimizes resources, enhances research quality, and prepares residents to tackle complex healthcare challenges (26). Similarly, interprofessional education fosters medical students' ability to work in collaborative teams, addressing multifaceted medical issues (27). Such collaborations yield benefits like shared research tasks, expanded information networks, varied perspectives, and innovative problem-solving (28).

#### Blended delivery of research curriculum

Blended leaning using technology is used in the delivery of the curriculum content across East Africa-campuses (coupled with in person workshops), in turn promoting access and wide coverage across the two countries (Kenya, Tanzania) where the residents are based. Blended learning, integrating face-to-face and online activities, is widely adopted in academic institutions to foster student autonomy, introspection, and research capabilities (22, 29). Effective integration of online and classroom components, supported by robust administrative systems, optimizes learning outcomes and enhances flexibility (30, 31).

#### Financial support and resources availability

At AKU, students can secure funding for research expenses through their departments, college seed funding, or the University Research Council (URC). Eligible residents, master's, and PhD students may receive up to US$ 2,000, 3,000, and 10,000 respectively from the URC (32). MCEA residents also benefit from US$ 1,000 in Dean's seed funding administered by the finance department, covering research costs such as materials and research assistants. This structured funding process ensures resources are used appropriately, easing financial burdens and allowing students to focus on research. Access to comprehensive library resources further supports academic growth (33).

#### Disseminating dissertation research results

Universities typically require researchers to disseminate their findings through publications and other channels (9). Medical specialists are expected to lead healthcare teams, maintain expertise in their fields, and critically assess emerging medical literature (34). Publishing research in peer-reviewed journals is crucial for professional growth in medical science (35). AKU residents share their research through departmental seminars, university-organized forums like FARs, and relevant local and international conferences. Presenting dissertation results at FARs or symposia is mandatory for sitting Part II examinations in the fourth year. Dissertation submission requires evidence of manuscript submission or acceptance for publication in peer-reviewed journals (18).

#### Research and dissertation quality assessment mechanisms

Quality management of dissertations is integral to postgraduate training excellence. Improving and controlling dissertation quality ensures the development of high-level talents (36). Institutions, including those in South Africa, use metrics like degree progress, dissertation quality, research communication skills, quality of the research training and research impact to assess postgraduate education and research (9). Initiatives such as faculty research coordinators, research tracking systems, and resident research coordinators enhance scholarly productivity during and after residency (37).

The MCEA has established robust procedures to monitor dissertation quality among residents, including ongoing curriculum improvements based on residents' and faculty feedback. Departmental Research Committees ensure adherence to timelines and maintain supervision records. The Scientific Research Committee oversees research compliance. Each student maintains comprehensive records of supervision activities. Dissertations are evaluated by two external examiners, endorsed by the Chief Internal Examiner (CIE). Successful approval requires endorsement by at least two examiners, a Turnitin similarity index ≤ 15%, and confirmation by the Associate Dean, PGME. Disputes are resolved by an impartial examiner appointed by the CIE, guided by SRC advice. Accepted dissertations include evidence of publication acknowledgment and undergo final review by the Dissertations Standard Sub-Committee before archiving (15).

### Residents' personal related factors

#### Protected research time

Protected training and research time coupled with mentorship from senior faculty, and funding can lead to residents' research productivity and accomplishment (38). The type of dedicated research time (blocked, longitudinal, or none) correlates directly with residents' research output (39). AKU provides structured four-year training with 96.5 contact hours (54 taught, 42.5 workshop), including up to 8 weeks of protected time for research activities like data collection and analysis (15). This dedicated time ensures residents can focus on their research, conduct thorough literature reviews, and produce high-quality work, crucial for successful dissertation completion and professional development.

#### Recognition of outstanding research

Upon completion of the dissertation, Departments normally submit a choice of the best dissertation in the departments to a committee of members selected by the University registrar. Upon a rigorous assessment by a team of more than three external reviewers the best dissertation receives recognition of outstanding work at the annual global convocation. These recognitions serve as a motivation for residents and their supervisors to put forth efforts to produce high quality research.

### Supervisor related factors

#### Supervision of residents dissertations

Effective dissertation supervision is crucial for students' success, shaping them into competent medical professionals (11, 40). Supervisors facilitate access to resources, review drafts, and offer constructive feedback, ensuring research quality, as well as shaping well-rounded and research-focused medical professionals (11). Supervisors are selected based on merit, including expertise in research specialty and methodology or a track record in scientific publication, to guide students effectively in research-related activities at AKU-MCEA.

#### Faculty development in dissertation supervision and research training

Despite the recognized significance of expertise in research supervision, research skills are not often prioritized during recruitment of faculty, focus is more on teaching and administrative duties. This limits faculty development in research supervision, including problem definition, methodology, data analysis, and publication. Comprehensive training programs for supervisor competencies are absent, despite their critical role in dissertation and research quality (40). Enhancing faculty academic skills cultivates expertise in supervision, enhancing their ability to guide dissertation projects effectively (33). AKU emphasizes a balanced approach for faculty, integrating teaching, research, and service, and fosters an environment for continuous learning to meet career goals and university vision. The MCEA's Department of Population Health organizes early career faculty development in dissertation supervision and research, spanning three modules over a year. This longitudinal training aims to equip faculty with supervisory skills and nurture them as independent researchers, culminating in grant submissions.

## Conclusion

The Dissertation-centric longitudinal approach to post-graduate level research methods training has proven to be a successful and transformative research training model. Having a dedicated research curriculum delivered longitudinally in tandem with expected research milestones, delivered through a systems-wide design of interactive didactic sessions complemented with mentored workshops delivered by multiple health professionals and a dedicated team of supervisors, in addition to having financial and resource support, protected research time, development of faculty skills in dissertation supervision and research, disseminating dissertation research policies and guidelines to faculty, staff and students/residents is likely to lead to timely completion of dissertation and accelerate dissemination of research findings.

## Data Availability

The data analyzed in this study is subject to the following licenses/restrictions: the data is administrative from students records that can be made available upon request through the corresponding author. Requests to access these datasets should be directed to muthoni.mwangi@aku.edu.
